# Concentrated Raw Fibers Enhance the Fiber-Degrading Capacity of a Synthetic Human Gut Microbiome

**DOI:** 10.3390/ijms22136855

**Published:** 2021-06-25

**Authors:** Alex Steimle, Mareike Neumann, Erica T. Grant, Jonathan D. Turner, Mahesh S. Desai

**Affiliations:** 1 Department of Infection and Immunity, Luxembourg Institute of Health, 4354 Esch-sur-Alzette, Luxembourg; Alexander.Steimle@lih.lu (A.S.); Mareike.Neumann@lih.lu (M.N.); Erica.Grant@lih.lu (E.T.G.); Jonathan.Turner@lih.lu (J.D.T.); 2 Faculty of Science, Technology and Medicine, University of Luxembourg, 4365 Esch-sur-Alzette, Luxembourg; 3 Odense Research Center for Anaphylaxis, Department of Dermatology and Allergy Center, Odense University Hospital, University of Southern Denmark, 5000 Odense, Denmark

**Keywords:** microbiota, microbiome, manipulation, fiber, diet, prebiotic, nutrition, dietary supplementation

## Abstract

The consumption of prebiotic fibers to modulate the human gut microbiome is a promising strategy to positively impact health. Nevertheless, given the compositional complexity of the microbiome and its inter-individual variances, generalized recommendations on the source or amount of fiber supplements remain vague. This problem is further compounded by availability of tractable in vitro and in vivo models to validate certain fibers. We employed a gnotobiotic mouse model containing a 14-member synthetic human gut microbiome (SM) in vivo, characterized a priori for their ability to metabolize a collection of fibers in vitro. This SM contains 14 different strains belonging to five distinct phyla. Since soluble purified fibers have been a common subject of studies, we specifically investigated the effects of dietary concentrated raw fibers (CRFs)—containing fibers from pea, oat, psyllium, wheat and apple—on the compositional and functional alterations in the SM. We demonstrate that, compared to a fiber-free diet, CRF supplementation increased the abundance of fiber-degraders, namely *Eubacterium rectale*, *Roseburia intestinalis* and *Bacteroides ovatus* and decreased the abundance of the mucin-degrader *Akkermansia muciniphila*. These results were corroborated by a general increase of bacterial fiber-degrading α-glucosidase enzyme activity. Overall, our results highlight the ability of CRFs to enhance the microbial fiber-degrading capacity.

## 1. Introduction

Diets prevalent in industrialized countries are characterized not only by high amounts of protein and fat, but also by a deficiency of plant-derived fibers [1]. These so-called “Western-style” nutritional habits are linked to altered and potentially disease-promoting properties of the intestinal microbiome [2], further suggesting that supplementation of such diets with prebiotic fibers might be beneficial for the host. The intestinal microbiome has a remarkable impact on susceptibility and progression of various intra- and extra-intestinal pathologies [2]. Thus, the targeted manipulation of the host’s microbiome may alleviate this risk and has recently received considerable attention [3]. In this context, plant-derived fibers are considered to be promising host-beneficial dietary supplements for microbiota modulation [4]. Health-beneficial impacts of fibers are either mediated by general physiological influences, maintaining the integrity of the mucus layer or by microbial fermentation into host-beneficial metabolites, such as short-chain fatty acids (SCFAs). SCFAs play a crucial role in maintaining barrier integrity and immune homeostasis [5], and soluble fibers represent a major source of these microbially produced metabolites [2]. 

Previously, we reported a causal role of fiber deprivation, increasing susceptibility towards enteropathogenic infections in a gnotobiotic mouse model containing a 14-member synthetic human gut microbiome (14SM) [6]. We demonstrated that a lack of dietary fiber resulted in a bloom of mucin-degrading commensals, such as *Akkermansia muciniphila,* leading to the excess degradation of the intestinal mucus layer, and subsequently, facilitated infection with *Citrobacter rodentium* [6]. These results further strengthen the connection between dietary fiber and gut microbial modulation. Moreover, our 14SM gnotobiotic model provides an attractive approach to validate the modulation of the gut microbiota with fiber supplementation using the basal fiber-free diet [6]. However, due to the complexity of the intestinal microbiome and the resulting individual responses, general recommendations on quantity, source or combinations of fiber supplements for consumption for humans remain vague [7,8]. 

Plant-derived fibers come in different chemical forms and structures, therefore providing distinct access for intestinal microbes to hydrolyze structure-specific glycosidic linkages. Here, we used dietary “concentrated raw fiber” (CRF) preparations from pea, oat, psyllium, wheat and apple to evaluate the detailed effects of fiber supplementation under strictly controlled conditions in our 14SM gnotobiotic mouse model. In contrast to purified fibers [9], CRFs are fiber concentrates, which are extracted and isolated from skeletal substances in a non-chemical, thermophysical process, thus providing a diverse polysaccharide composition. Of note, the wheat CRF used was previously shown to increase fecal bulking in a randomized controlled human study [10], while the psyllium CRF was associated with an increased overall SCFA production [11] in an in vitro system. We evaluated the in vivo effects of these CRFs on the relative abundances of the 14SM constituent strains, the emerging activities of bacterial glycan-degrading enzymes and the associated concentrations of different SCFA. Furthermore, we performed extensive correlation analyses to evaluate potential inter-microbial influences in response to fiber supplementation and thus better understand community-shaping properties of such dietary modulation. 

## 2. Results

### 2.1. Experimental Setup to Study the Specific Effects of Concentrated Raw Fibers on Composition and Function of A 14-Member Microbial Community in Mice

**Figure 1:**
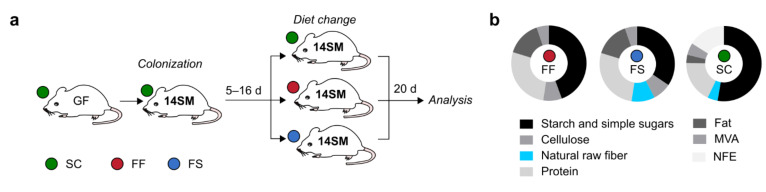
Experimental outline to study effects of concentrated raw fibers on a 14-member microbial community. (**a**) Experimental outline. Germ-free (GF) C57BL/6N (*n* = 15), raised and maintained on a standard mouse chow (SC) were colonized with the 14SM community. Five to sixteen days after colonization, mice were either continued to be fed a SC diet (*n* = 7) or switched to a fiber-free (FF; *n* = 4) or a fiber-supplemented (FS, *n* = 4) diet. Twenty days after diet switch, cecal and fecal samples were harvested for analyses. (**b**) Composition in % (*w*/*w*) of the diets; MVA = Minerals, vitamins, and ash; NFE = Nitrogen-free extracts.

Germ-free (GF) C57BL/6N mice were raised and maintained under gnotobiotic conditions on a standard mouse chow (SC). At the age of six to eight weeks, mice were colonized via intragastric gavage with a synthetic microbiota consisting of 14 human commensals (14SM), as described previously [6]. Strains of this 14SM community represent the five dominant phyla of the human intestinal microbiota and provide important core metabolic function [6]. Five to sixteen days after the initial gavage, mice were either switched to a fiber-free (FF) diet or a fiber-supplemented (FS) diet (Figure 1a) containing CRFs (VITACEL^®,^ J. Rettenmaier und Söhne (JRS, Rosenberg, Germany)) derived from pea, oat, psyllium, wheat and apple. As controls, seven mice were maintained on a SC diet. Before the diet switch, we confirmed the proper colonization of all animals with the 14SM community by strain-specific qPCR from fecal samples, as described previously [6]. Twenty days after the diet switch (feeding period), mice fed all three different diets were sacrificed and contents of the cecum and colon were harvested for downstream analyses. As we aimed to determine the direct impact of fiber supplementation on microbiota composition and function in a tightly controlled gnotobiotic setting, we designed the FF and FS diets with the aim of providing an isocaloric composition as well as an identical formulation among these two diets, with the exception of the non-cellulose complex fiber amount (Figure 1b).

Thus, to generate the FS diet, we reduced the dextrose content in the FF diet by an amount corresponding to 10% of the total weight and replaced it by the same amount of a concentrated raw fiber mix. The fiber mix in the FS diet consisted of equal amounts of CRF preparations obtained from pea, oat, psyllium, wheat and apple (2% (*w*/*w*) each). The five different CRF preparations contained an average fiber length within a two-digit µm range and the fiber content (*w*/*w*) in these preparations ranged from 55% (apple) to 97% (wheat) (Table 1). Importantly, the ratio of insoluble-to-soluble fibers (I/S-ratio) differed significantly among the different preparations, with an I/S ratio of 34 in the case of the pea preparation to an I/S ratio of 0.2 for the psyllium preparation. Of note, the apple preparation contained 9% (*w*/*w*) pectin. The SC diet contained 3.9% fibers from naturally milled fibers, while contents of protein and fat were considerably lower compared to both, the FF and FS diets (Figure 1b).

### 2.2. Increase in Relative Abundance of Certain Fiber-Degrading Commensals in Response to Dietary Fiber Supplementation

**Figure 2:**
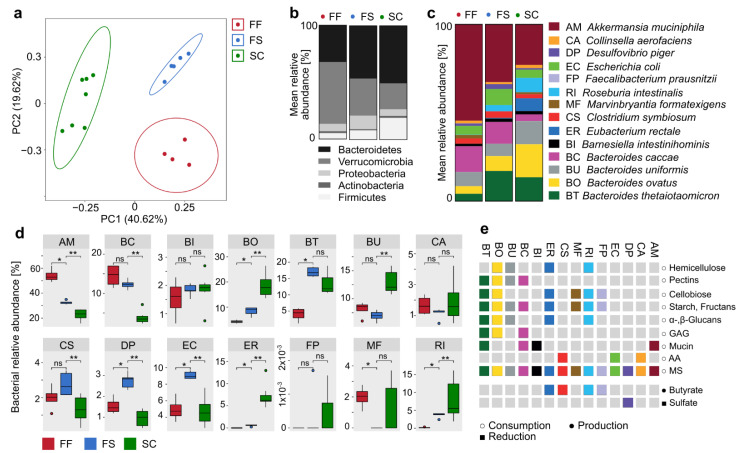
Compositional changes within a synthetic microbiota in response to dietary fiber supplementation. (**a**) PCA plot of the microbiota composition as calculated with the “prcomp” function within the R package “stats”, using 16S rRNA gene sequencing data from fecal samples and based on a Euclidian distance matrix. (**b**) Mean relative abundances of the 14SM microbial community on a phylum level. (**c**) Left panel: Mean relative abundance of each constituent strain of the 14SM community in mice fed either FF, FS or a SC diet. Right panel: Names of the 14SM-constituent strains and their two-letter abbreviations. (**d**) Tukey boxplots of the relative abundance of each strain; outliers shown as circles; statistics: Wilcoxon rank sum test performed with the “compare_means” function within the R package “ggpubr”; *: *p* < 0.05, **: *p* < 0.01. (**e**) Metabolic characteristics of each strain as determined in detail previously [6]; colored boxes represent metabolic activity related to the indicated substances; grey boxes represent no metabolic activity. GAG = glucosaminoglycans, AA = amino acids, MS = monosaccharides. AM: *A. muciniphila*, BC: *B. caccae*, BI: *B. intestinihominis*, BO: *B. ovatus*, BT: *B. thetaiotaomicron*, BU: *B.* uniformis, CA: *C. aerofaciens*, CS: *C. symbiosum*, DP: *D. piger*, EC: *E. coli*, ER: *E. rectale*, FP: *F. prausnitzii*, MF: *M. formatexigens*, RI: *R. intestinalis*.

At the end of the 20-day feeding period with the three diets, the microbiota composition was analyzed in fecal samples using 16S rRNA gene sequencing, revealing different clustering of each of the three groups (Figure 2a). These findings not only highlight the overall impact of diet on the microbiota composition, but also the specific and considerable effect of CRF supplementation. On a phylum level, we determined a significant increase in the abundance of Bacteroidetes (*p* = 0.0046; *t*-test) and a decrease in Firmicutes (*p* = 0.0149; *t*-test) (Figure 2b) in FS-fed mice compared to their FF-fed counterparts. On a strain-level, we detected significantly different relative abundances of 8 of the 14 community members in FS-fed mice compared to the FF-fed control mice (Figure 2c,d). Specifically, we detected significantly lower abundances of *A. muciniphila* and *M. formatexigens* (see Figure 2c for strain abbreviations) in FS-fed mice compared to their FF-fed counterparts, while the relative abundances of *E. coli*, *D. piger*, *E. rectale*, *B. ovatus*, *R. intestinalis* and *B. thetaiotaomicron* were significantly increased (Figure 2d) in response to CRF supplementation. 

However, we also detected significant differences in the relative abundances of nine strains when comparing the FS-fed to the SC-fed mice. Since the SC diet also contains natural fiber, albeit in a non-concentrated form and in lower amounts, these differences could not be rooted in presence of fibers alone and might be a result of different sources of fibers or the distinct protein and fat content. Since we were particularly interested in strain-specific changes in response to fiber supplementation and the emerging effects on microbiota function, we associated strain-specific changes between the FS- and FF-fed mice with the metabolic potential of the respective strains to grow on a suit of mono- and polysaccharides as determined previously using carbohydrate in vitro growth assays [6] (Figure 2e). This association revealed a decreased relative abundance of a mucin specialist, *A. muciniphila* (Figure 2d), which is in line with previous findings that fiber deprivation results in overgrowth of this particular strain [6] leading to decreased mucosal barrier integrity. This finding further highlights the inverse correlation between relative *A. muciniphila* abundance in the colon and dietary fiber intake. Of note, increased abundances of *A. muciniphila* were associated with various pathologies in human studies [12,13,14,15,16,17], supporting the idea that, in addition to promoting microbiota-mediated SCFA production, fiber supplementation considerably contributes to the maintenance of mucosal barrier integrity by preventing excess mucus degradation. 

Furthermore, fiber supplementation resulted in significantly increased relative abundances of *B. thetaiotaomicron*, *E. rectale*, *R. intestinalis* and *B. ovatus* (Figure 2d), which share the capability to metabolize a broad variety of plant-derived polysaccharides, such as starch, cellobiose and α- and/or β-glucans (Figure 2e) as previously confirmed with a carbohydrate in vitro utilization assay [6]. Thus, the ability to degrade α- and/or β-glucans promoted commensal growth under CRF-supplemented conditions, probably due to such glucans being major components of the CRF preparations [18]. Importantly, not all of the strains capable of metabolizing complex polysaccharides, such as *B. uniformis*, were increased in response to supplementation with the selected fiber formulation (Figure 2d), indicating strain-specific effects of supplementation with the chosen CRF supplements. In general, intensified fiber consumption by the microbiome is associated with increased intestinal H_2_ levels, which can exhibit disadvantageous effects on the host [19]. Thus, the increased abundance of *D. piger* in FS-fed mice might have a counter-regulating effect given the H_2_-consuming properties of this bacterium [20]. Since *E. coli* is not a fiber fermenter [6], its increased abundance is very likely a secondary effect due to changed abundances of microbes that are directly affected by fiber supplementation, resulting in altered microenvironments or nutrient availability.

### 2.3. Inter-Bacterial Relations in Relative Abundance within the 14-Member Microbial Community

**Figure 3:**
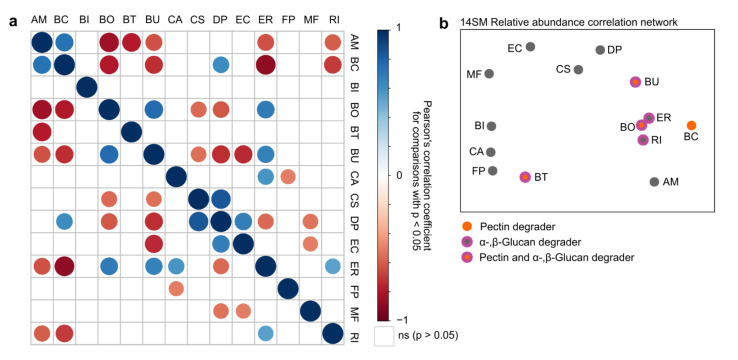
Correlations between relative abundances of 14SM community members. (**a**) Pairwise correlation between relative abundances of all strains as determined by using 16S rRNA gene sequencing data from all mice across all groups; Analysis performed using the “rcorr” function of the R package “Hmisc” and visualized using the “corrplot” function. Colored circles depict statistically significant correlations (*p* < 0.05); empty squares represent non-significant correlation independent of the determined correlation coefficient *R*; color intensity and circle size vary depending on the Pearson correlation coefficient *R,* with *R* = 1 (positive correlation) and *R* = −1 (negative correlation) displayed with maximal circle size and color intensity. (**b**) Correlation network analysis using the “network_plot” function in the R “rcorr” package. Each circle represents one of the 14SM strains. Circles are positioned by multidimensional scaling of the absolute values of the correlations shown in panel a) to highlight correlation clusters in a two-dimensional graph. AM: *A. muciniphila*, BC: *B. caccae*, BI: *B. intestinihominis*, BO: *B. ovatus*, BT: *B. thetaiotaomicron*, BU: *B. uniformis*, CA: *C. aerofaciens*, CS: *C. symbiosum*, DP: *D. piger*, EC: *E. coli*, ER: *E. rectale*, FP: *F. prausnitzii*, MF: *M. formatexigens*, RI: *R. intestinalis*.

To further investigate such potential inter-microbial influences and dependencies in response to CRF supplementation, we performed pairwise correlation analyses of all strains within each individual and across all groups (Figure 3a). All correlation analyses were performed using the “rcorr” function within the R package “Hmisc” and visualized using the “corrplot” package. While the relative abundances of some strains, such as *B. intestinihominis*, *F. prausnitzii* and *B. thetaiotaomicron*, provided little to no correlation with any of the other community members, certain bacteria, such as *A. muciniphila*, *B. caccae*, *B. ovatus*, *B. uniformis*, *D. piger* or *E. rectale*, significantly correlated with multiple other strains (Figure 3a). These findings indicate a high inter-microbial dependency of *A. muciniphila*, *B. caccae*, *B. ovatus*, *B. uniformis*, *D. piger* or *E. rectale* with other strains within the 14SM community, suggesting that the relative abundance of these strains was either strongly dependent on the overall microbiota composition or, conversely, are major influencers of the remaining microbiota in response to certain environmental changes, such as dietary supplementation. In addition to *B. thetaiotaomicron*, all other α-and β-glucan metabolizing microbes (*B. ovatus*, *B. uniformis*, *E. rectale* and *R. intestinalis*) provided significantly positive correlations with each other (Figure 3a; Pearson correlation coefficient *R* > 0), suggesting that there was no nutrient competition for these polysaccharides between these strains. Interestingly, *B. caccae*, which is able to metabolize pectin but not α- and β-glucans, provides a strong negative correlation to all of the α- and β-glucan degraders. Given the overall pectin concentration in the FS diet of roughly 0.2% (*w*/*w*) (Table 1), this suggested a potential competition for pectin with those α- and β-glucan degraders, which share the ability to metabolize pectin.

To better illustrate such potential inter-microbial correlations, we performed a correlation network analysis (Figure 3b), employing the “network_plot” function in the R “rcorr” package to highlight clusters of the correlations shown in Figure 3a. In this plot, variables undergo multidimensional clustering using the absolute values of the correlations, where tightly clustered variables exhibit similar relationships with the other variables (Figure 3b). Figure 3b demonstrates that strains metabolizing α- and β-glucans are clustering strongly together. Interestingly, *B. caccae* (pectin degrader) and *A. muciniphila* (mucin degrader) also fall into the same correlation cluster (Figure 3b) through their significant negative correlation with the glucan degraders (Figure 3a), suggesting that the decreased relative abundance of *A. muciniphila* under fiber-supplemented conditions (Figure 2d) is a secondary effect due to the bloom of fiber-fermenting microbes, while *B. caccae* remains unaffected in its relative abundance in FS-fed mice (Figure 2d), while it is strongly decreased in SC-fed mice.

### 2.4. Concentrated Raw Fiber Supplementation Is Associated with Changes in Fecal Bacterial Glycan-Degrading Enzymes

**Figure 4:**
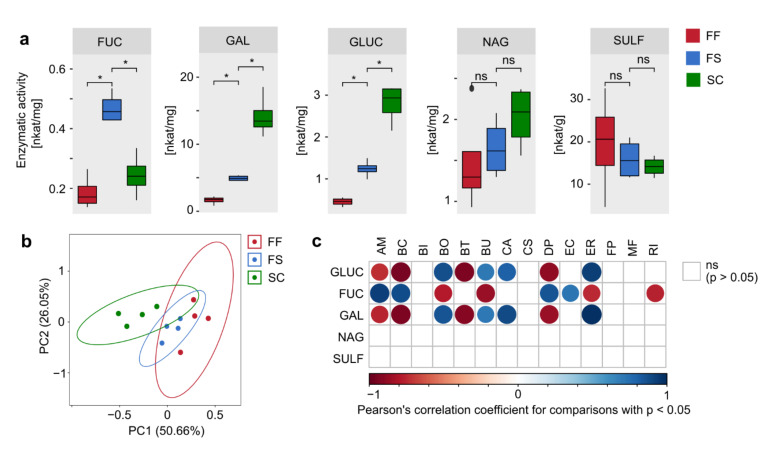
Concentrated raw fiber supplementation results in increased activity of bacterial fiber-degrading enzymes. (**a**) Tukey boxplots of enzymatic activities of bacterial glycan-degrading enzymes in fecal samples, normalized on the amount of total fecal protein; FUC: α-fucosidase, GLUC: β-glucosidase, GAL: α-galactosidase, NAG: β-*N*-acetlyglucosaminidase, SULF: sulfatase; statistics: Wilcoxon rank sum test performed with the “compare_means” function within the R package “ggpubr”. *: *p* < 0.05. (**b**) PCA plot of the glycan-degrading enzyme activity pattern of FF-, FS- and SC-fed mice as calculated with the “prcomp” function within the R package “stats”, using data sets shown in (**a**) and based on a Euclidian distance matrix. Visualization using the “autoplot” function. (**c**) Pairwise correlation between glycan-degrading enzyme activities and the relative abundance of 14SM strains from all mice across all groups. Analysis performed using the “rcorr” function of the R package “Hmisc” and visualized using the “corrplot” function. Colored circles depict statistically significant correlations (*p* < 0.05); empty squares represent non-significant correlation; color intensity and circle size vary depending on the Pearson correlation coefficient *R,* with *R* = 1 (positive correlation) and *R* = −1 (negative correlation) displayed with maximal circle size and color intensity. AM: *A. muciniphila*, BC: *B. caccae*, BI: *B. intestinihominis*, BO: *B. ovatus*, BT: *B. thetaiotaomicron*, BU: *B.* uniformis, CA: *C. aerofaciens*, CS: *C. symbiosum*, DP: *D. piger*, EC: *E. coli*, ER: *E. rectale*, FP: *F. prausnitzii*, MF: *M. formatexigens*, RI: *R. intestinalis*.

Given these fiber supplementation-mediated changes of microbial abundances (Figure 2 and Figure 3), we evaluated the functional outcomes of these compositional alterations. Thus, we determined the enzymatic activity of certain bacterial enzymes in fecal pellets that are involved in either the fermentation of fiber-derived polysaccharides or the degradation of host-secreted mucin glycans, which were previously reported to be inversely associated with the amount of dietary fiber consumed [6]. The enzymes β-glucosidase (GLUC) and α-galactosidase (GAL) primarily target glycosidic linkages present in plant fiber-derived polysaccharides, with β-glucosidase being a crucial enzyme for hydrolyzing linkages in β-glucans [6]. Conversely, α-fucosidase (FUC), sulfatase (SULF) and β-*N*-acetylglucosaminidase (NAG) catalyze reactions involved in mucin glycan degradation [6]. While fecal activities of SULF and NAG remained unaffected by fiber supplementation, we detected significantly increased activities of GLUC and GAL in FS-fed mice compared to FF-fed mice, albeit FS-fed mice provided significantly lower GAL and GLUC activities as compared to SC-fed controls (Figure 4a). This indicates that the source of fibers and their fine-scale composition seems to be more important to mediate functional outcomes of the microbiome than the amount of CRFs alone. Surprisingly, we also detected a significant increase in FUC activities in FS-fed mice (Figure 4a). This may be due to the presence of certain glycans in the FS diet harboring an alpha-1,6-linked fucose residue joined to the reducing end of an *N*-acetylglucosamine moiety, which is absent in the other diets. 

The overall glycan-degrading enzyme activity pattern, as determined by principal components analysis (PCA) using activity data of all determined enzymes, revealed a different clustering between FF- and SC-fed mice only, while FS-fed mice provided an intermediate activity pattern (Figure 4b). The activity of GLUC and GAL exhibited a strong positive correlation with the relative abundance of the fiber-degrading strains *B. ovatus*, *B. uniformis* and *E. rectale*, but also with *C. aerofaciens* (Figure 4c), which might benefit from released monosaccharides from polysaccharide degradation catalyzed by other strains. As expected, FUC activity exhibited a strong positive correlation with the relative abundance of mucin-glycan-degrading *A. muciniphila*, but also with *D. piger*, *B. caccae* and *E. coli* (Figure 4c). While *B. caccae* is mucin generalist, meaning that it is also capable of mucin degradation (Figure 2e), based on our previous work [6], *E. coli* is neither a mucin glycan-degrading nor a fiber-degrading commensal (Figure 2e). Although correlation analyses suggest that increased FUC activity in FS-fed mice are associated with *E. coli*, we could, so far, not confirm FUC expression in *E. coli*, and this finding might be a non-causal, correlative artifact due to secondary effects.

### 2.5. Changes of Bacterial Glycan-Degrading Enzyme Activities Are Interlinked with a Specific Short-Chain Fatty Acid Production Profile

**Figure 5:**
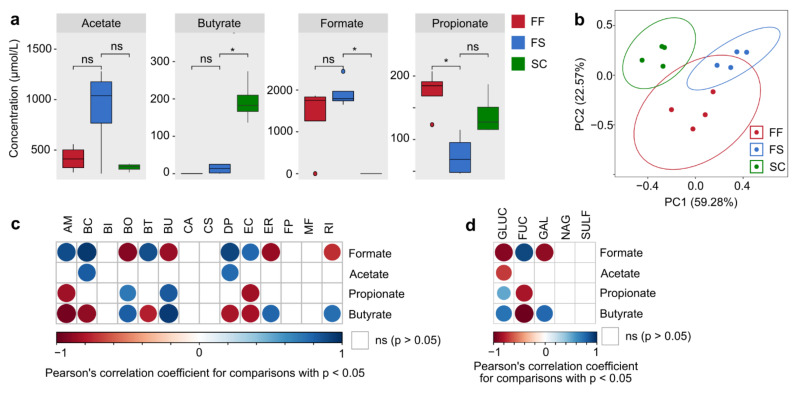
Functional changes of bacterial enzyme activity are interlinked with a specific short-chain fatty acid profile. (**a**) Tukey boxplots of short-chain fatty acid (SCFA) concentrations in cecal contents, normalized on the amount of cecal matter; statistics: Wilcoxon rank sum test performed with the “compare_means” function within the R package “ggpubr”, * *p* < 0.05. (**b**) PCA plot of the cecal SCFA concentration pattern of FF-, FS- and SC-fed mice as calculated with the “prcomp” function within the R package “stats”, of data sets shown in (**d**) and based on a Euclidian distance matrix. Visualisation using the “autoplot” function. (**c**) Pairwise correlation between cecal SCFA concentrations and the relative abundance of 14SM strains (**d**) Pairwise correlation between cecal SCFA concentrations and glycan-degrading enzyme activities from all mice across all groups; characteristics of correlation matrix are identical to panel (**a**). (**c**,**d**) Analysis performed using the “rcorr” function of the R package “Hmisc” and visualized using the “corrplot” function. Colored circles depict statistically significant correlations (*p* < 0.05); empty squares represent non-significant correlation; color intensity and circle size vary depending on the Pearson correlation coefficient *R*, with *R* = 1 (positive correlation) and *R* = −1 (negative correlation) displayed with maximal circle size and color intensity. AM: *A. muciniphila*, BC: *B. caccae*, BI: *B. intestinihominis*, BO: *B. ovatus*, BT: *B. thetaiotaomicron*, BU: *B. uniformis*, CA: *C. aerofaciens*, CS: *C. symbiosum*, DP: *D. piger*, EC: *E. coli*, ER: *E. rectale*, FP: *F. prausnitzii*, MF: *M. formatexigens*, RI: *R. intestinalis*, GLUC: β-glucosidase, GAL: α-galactosidase, FUC: α-fucosidase, NAG: β-*N*-acetylglucosaminidase, SULF: sulfatase.

In addition to other features, microbe-mediated fiber degradation results in the production of short-chain fatty acids (SCFAs) [21]. Since SCFAs provide important beneficial effects for the host [5,22], we next investigated whether the fiber degradation-associated enzyme activity pattern in FS-fed mice resulted in altered SCFA production. While cecal concentrations of acetate and formate did not differ significantly between the FF- and FS-fed mice, propionate concentrations in cecal contents were significantly lower in the FS-fed mice compared to their FF-fed counterparts (Figure 5a). In line with this, PCA analysis of the overall SCFA production pattern between the three groups revealed that CRF supplementation did not result in a significantly different SCFA production compared to FF-fed mice, while the SC-mediated SCFA production was significantly different from the FF diet (Figure 5b). In contrast to the SC-fed control group, the concentration of the main host-modulatory SCFA, butyrate [23], did not increase in FS-fed mice compared to FF-fed mice (Figure 5a). The most important butyrate producers within the 14SM community are *F. prausnitzii*, *R. intestinalis*, *C. symbiosum* and *E. rectale* [6] (Figure 2e). 

Although the relative abundances of *R. intestinalis* and *E. rectale* were significantly higher in the FS-fed mice compared to the FF-fed mice (Figure 2d), the abundances of these strains were significantly lower compared to SC-fed control mice, which provided the highest butyrate concentrations among the three groups. Thus, the relative abundance of *R. intestinalis* and *E. rectale* appear to be predictors of butyrate concentration in 14SM-colonized mice, due to their strong positive correlation with corresponding butyrate levels (Figure 5c) and their relative abundances in FS-fed mice was probably not elevated enough to translate into significant increases in butyrate and propionate concentrations compared to FF-fed mice. Additionally, the relative abundance of *B. ovatus* also correlated positively with butyrate concentrations (Figure 5c). However, this correlation is probably rooted in non-butyrate related inter-microbial interactions, since this strain is not known to be a main butyrate producer within the 14SM community. Furthermore, while butyrate concentrations exhibited positive correlation with GLUC and GAL activities, propionate only correlated positively with GLUC and formate with FUC activities (Figure 5d).

**Figure 6:**
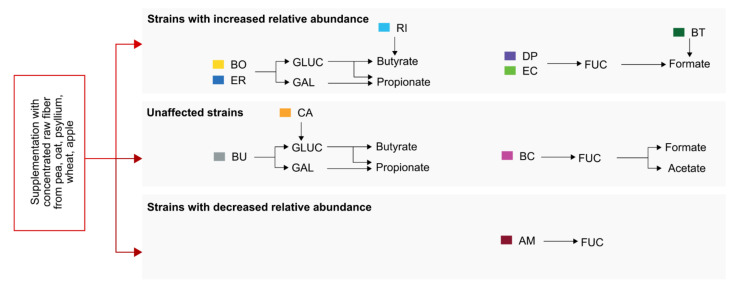
Summary of correlations between relative abundances of 14SM strains, glycan-degrading enzyme activity and SCFA production in response to fiber supplementation. Strains that are significantly increased in FS-fed mice compared to FF-fed mice are depicted in the upper panel, while unaffected strains are shown in the middle panel and strains with decreased relative abundance in the lower panel. Arrows only show significant (*p* < 0.05) positive (0 < *R* < 1) correlations as determined in Figure 3, Figure 4 and Figure 5. AM: *A. muciniphila*, BC: *B. caccae*, BO: *B. ovatus*, BT: *B. thetaiotaomicron*, BU: *B. uniformis,* CA: *C. aerofaciens*, DP: *D. piger*, EC: *E. coli*, RI: *R. intestinalis*, GLUC: β-glucosidase, GAL: α-galactosidase, FUC: α-fucosidase.

In summary, given the strong effects of fiber supplementation on the relative abundances of certain fiber degraders (Figure 2c,d) and the associated increase in bacterial fiber-degrading enzyme activities (Figure 4a), the non-significant SCFA levels compared to FF-fed mice (Figure 5a) were somewhat unexpected but in line with the relatively decent increase of GLUC and GAL activities compared to the FF-fed mice. Combining the data from Figure 2, Figure 3, Figure 4 and Figure 5 suggests the presence of two independent functional correlation pathways connecting the 14SM community with glycan-degrading enzyme activities and SCFA production (Figure 6). While we found a strong positive correlation between GLUC and GAL activity with butyrate and propionate levels, FUC activity correlated with the production of acetate and formate. In addition to the ability of the host to metabolize certain amino acids into formate, it can also be produced as a by-product of metabolic activities of intestinal commensals [24]. Importantly, elevated concentrations of formate were previously reported to be a signature feature of inflammation-associated microbiome dysbiosis in a mouse model of colitis [25] and was associated with increased abundances of commensal *E. coli* strains, which is in line with our correlation analyses (Figure 5c or Figure 6). 

Supplementation of the FF diet with the chosen mix of CRFs derived from pea, oat, psyllium, wheat and apple did result in an increased relative abundance of some, but not all strains that were found to correlate with the GLUC/GAL-associated pathway. Meanwhile, *D. piger* and *E. coli*, which correlate with the FUC-associated pathway, were increased. It is worth noting, that such identified correlations do not necessarily equate with causal connections within these pathways. For example, *B. uniformis* strongly correlated with GLUC and GAL activities as well as with cecal butyrate concentrations, although this species is not a known butyrate producer. Consequently, this strain might be important to support the butyrate production of other strains via yet unknown mechanisms. 

## 3. Discussion

A lack of fiber intake is commonly associated with decreased microbial diversity in the gut [26] as well as with increased concentrations of metabolites that can be harmful to the host [27]. Thus, supplementing a fiber-deprived, Western-style diet [2] appeared as a reasonable strategy to restore or even boost host-beneficial properties of a given individual’s indigenous microbiome. Although this approach seems trivial at first glance, it launches several challenges concerning the dose or source of fiber to be consumed by a certain individual, fitting the preconditions and needs of a specific microbiome composition. Although various human studies demonstrated beneficial effects of general fiber supplementation in most, but not all, study participants (reviewed in [28]), personalized and more tailored approaches are rare. Additionally, in-depth analyses of microbiome-specific effects of fiber supplementation on inter-microbial interactions and the resulting functional outcomes are difficult to conduct due to the complexity of the microbiome and the multitude of potential inter-microbial influences. 

Thus, we aimed to investigate such interconnections and functional outcomes in a gnotobiotic mouse model with a standardized microbiome consisting of 14 human commensal bacteria strains. These commensals comprise the five most abundant bacterial phyla in the human host and provide all core metabolic functions. Importantly, their ability to consume certain poly- and monosaccharides, as well as their capacity to produce SCFAs has been assessed previously [6]. We could demonstrate that most, but not all, fiber-degrading commensals within this community provided increased relative abundances in response to supplementation of a fiber-deprived diet with a mix of CRFs obtained from pea, oat, psyllium, wheat and apple. This was particularly the case for α- and β-glucan degrading commensals, while commensals capable of hydrolyzing pectin, which is prevalent in the added apple preparation, were not positively affected. Although the relative abundances of fiber-degrading commensals and the activities of enzymes involved in bacteria-mediated fiber degradation increased significantly in response to fiber supplementation, this did not translate into a more host-beneficial SCFA production pattern. Our correlation network analyses suggest dynamic interconnections in response to fiber supplementation between the 14 constituent strains and reveal the importance of yet unidentified inter-microbial interactions to exhibit beneficial properties. Previous studies have routinely used purified fiber supplements in rodent systems to show a positive impact on the generation of SCFAs [29]. However, one needs to be cautious about the high amount of fibers used in the rodent diets and the translatability of such amounts to human hosts. 

Given the far more complex microbiome composition and associated microbial interconnections and dependencies in the human gut, this highlights the challenges in designing personalized dietary recommendations for the benefit of the host. A pioneering study to address these points investigated the effects of four different fiber supplements on microbial diversity and emerging SCFA production in a human trial [30]. Among other findings, applied dietary fiber interventions were specific and limited to a few taxa within each participant, which, however, translated into a relatively consistent SCFA production pattern across the participants receiving the same supplements [30]. However, only ten participants were recruited for each cohort, which seems insufficient to make generalized statements on suitability of these supplements for a larger pool of individuals.

In summary, our findings demonstrate that supplementation of a fiber-free diet with a mix of CRFs resulted in significant changes to the intestinal microbiome structure and activity. While some, but not all, fiber-degrading commensals provided increased abundances in response to fiber supplementation, abundances of the mucin-specialist *A. muciniphila* were significantly decreased. Interestingly, increased abundances of this species were detected in multiple sclerosis patients [12,13,14,31] and were implicated in the increased susceptibility towards enteropathogenic infections in the same 14SM mouse model as used in this study [6]. However, other studies report on strong host-beneficial effects of this species [17,32], in some cases classifying *A. muciniphila* as “probiotic” [33]. These findings, which appear contradictory at first, might be rooted in different microbiome-mediated mechanisms of disease pathology, the considerable diversity among different commensal *A. muciniphila* strains [34] or in the complex inter-microbial influences within a given microbiome, which we observe even in a reduced community of only 14 strains. Thus, these factors might determine either the health-beneficial or disease-promoting properties of *A. muciniphila*. Given the specialization of *A. muciniphila* on degrading mucin-associated glycans, increased abundances of this species might result in excess mucus layer degradation under certain circumstances. Interestingly, impairment of the intestinal mucus layer integrity was already suggested to be involved in the pathology of ulcerative colitis [35,36]. The mucus layer represents a key component of the intestinal mucosal barrier, and increased mucosal barrier permeability is supposed to be a contributing factor to pathophysiology of autoimmune diseases [37]. However, the specific role of microbiome-mediated mucus degradation in this process is yet unclear and a potential pivotal contribution of particular commensal species remains to be elucidated. Either way, we demonstrate that dietary habits crucially impact the activity of bacterial mucin glycan-degrading enzymes, possibly resulting in altered mucosal barrier integrity. Thus, over-focusing on SCFA production should be avoided when assessing host-influencing properties of a diet-modulated microbiome. On the other hand, other beneficial properties of a fiber-modulated microbiome, such as regulation of the mucus turnover, could be taken into account. 

## 4. Materials and Methods

### 4.1. Mouse Experiments

Germ-free (GF) female C57BL/6N mice were originally purchased from Taconic Biosciences, Germany. The animals were bred and housed inside the local germ-free facility of the University of Luxembourg. Aerobic and anaerobic microbial culturing of fecal samples was used to confirm the GF status of mice. For ethical aspects of the performed animal experiments, see “Institutional Review Board Statement” below. Mice were raised and maintained under gnotobiotic conditions on a standard mouse chow (SC). The animals were kept in ISO-cages with a maximum of 5 mice per cage and colonized at the age of six to eight weeks via intragastric gavage with a synthetic microbiota consisting of 14 different human commensals (14SM), as described previously [6]. Five to sixteen days after initial gavage, after 14SM colonization confirmation via qPCR, mice were either switched to a fiber-free (FF) diet, a fiber-supplemented (FS) diet or remained on the SC diet as a control group. All diets and water were provided in sterile conditions ad libitum. The well-being of all animals was evaluated, and fecal samples were collected once per week. Twenty days after diet switch, mice fed all three different diets were sacrificed and contents of the cecum and colon were harvested for downstream analyses.

### 4.2. Animal Diets

The fiber-free (FF) diet and the fiber-supplemented (FS) diet were manufactured by SAFE diets (SAFE, Augy, France) and were synthesized according to a modified version of the Harlan TD.08810 diet. The composition of the FF diet was (per 1000 g of total diet weight): 269 g casein, 4 g L-cystine, 444.235 g dextrose monohydrate, 75 g corn oil, 75 g lard, 80 g cellulose, 35 g mineral mix (AIN-93G-MX (94046)), 15 g vitamin mix (AIN-93-VX (94047)), 2.75 g cholin bitatrate and 15 mg TBHQ. The composition of the FS diet was (per 1000 g): 269 g casein, 4 g L-cystine, 344.235 g dextrose monohydrate, 75 g corn oil, 75 g lard, 80 g cellulose, 35 g mineral mix (AIN-93G-MX (94046)), 15 g vitamin mix (AIN-93-VX (94047)), 2.75 g cholin bitatrate, 15 mg TBHQ, 20 g VITACEL pea fiber EF 150, 20 g VITACEL oat fiber, HF401-30, 20 g VITACEL psyllium husk fiber P-95, 20 g VITACEL wheat fiber WF-101 and 20 g VITACEL organic apple fiber AF400-30. All VITACEL fibers were kindly provided by J. RETTENMAIER & SÖHNE GMBH + CO KG, (JRS, Rosenberg, Germany). The standard chow is the mouse chow used in the local gnotobiotic facility, which was also manufactured by SAFE diets (version A04, product code U8233G10R). All diets were sterilized using 9 kGy gamma irradiation. 

### 4.3. Culturing and Colonization of Germ-Free Mice with Synthetic Microbiota

Culturing of all 14 bacterial strains of the synthetic microbiota (SM) and subsequent colonization of germ-free C57BL/6 mice was performed as described in detail previously [38]. In brief, all strains were cultured in a modified yeast- and short-chain fatty acid-containing culture medium (mYCFA), which was based on a previously published recipe [39]. However, its composition was adapted to fit the specific needs of the bacterial strains used in this study. Thus, mYCFA did not contain maltose and cellobiose, but contained N-acetyl-D-glucosamine to support growth of the mucin-specialist *A. muciniphila*. Furthermore, the concentration of sulfate ions was increased 46-fold and sodium lactate was added to support growth of *Desulfovibrio piger* [38]. Culturing of all strains was started 3 d before initial gavage by inoculation of 50 µL cryo-preserved bacterial cultures into 3 mL of oxygen-reduced mYCFA. Cultures were diluted daily by factor 100 in mYCFA if OD_600_ was higher than 0.4. The final gavage mix consisted of equal volumes of each bacterial culture, which were grown to an OD between 0.5 and 2.0 [38]. As described in detail elsewhere [38], this way of preparing the gavage mix results in reproducible colonization of GF mice with comparable relative abundances of a given strain across different experiments.

### 4.4. Isolation of Bacterial DNA from Mouse Fecal Samples

Collected mouse fecal samples were stored at –20 °C until processing for bacterial DNA extraction. Isolation of bacterial DNA from these fecal samples was performed using a phenol:chloroform:isoamyl alcohol (25:24:1)-based approach, followed by purification of DNA with the QIAGEN DNeasy Blood & Tissue kit, as previously described in detail [6,38]. In brief, 500 µL of “Buffer A” (0.2 M NaCl, 0.2 M Trizma base, 20 mM EDTA pH 8), 210 µL 20% (*w*/*v*) SDS (pH 5.2) and 500 µL phenol:chloroform:isoamyl alcohol (25:24:1) (pH 8.0) were added to one fecal sample of approx. 20–40 mg. After adding 250 µL of acid-washed glass beads (212–300 μm) to this mixture, samples were subjected to bead-beating on the highest frequency (30 Hz) for 3 min using a bead mill. After centrifugation at 18,000× *g* and 4 °C for 3 min, the aqueous phase was harvested and 500 µL of a phenol:chloroform:isoamyl alcohol (25:24:1) mix was added. After mixing by tube inversion, samples were centrifuged again at 18,000× *g* and 4 °C for 3 min. The aqueous phase was harvested and 500 µL of 100% chloroform was added to the harvested aqueous phase followed by mixing through tube inversion. Samples were centrifuged at 18,000× *g* and 4 °C for 3 min, followed by another aqueous-phase harvesting. Next, 60 µL 3 M NaCl (pH 5.5) and 600 µL 100% iso-propanol were added and incubated for 1 h at −20 °C for DNA precipitation. After centrifugation at maximum speed and 4 °C for 20 min, the supernatant was discarded, and the pellet was resuspended in 1 mL 70% ethanol. After centrifugation for 3 min at max speed, the supernatant was removed, and the pellet was dried. The dry pellet was resuspended in 100 µL nuclease-free water and subjected to further DNA purification using the QIAGEN DNeasy Blood & Tissue kit according to the manufacturer’s instructions.

### 4.5. Illumina 16S rRNA Gene Sequencing and Data Analysis

This protocol uses dual-index primers to amplify the V4 region of the 16S rRNA gene [40]. For each plate, ZymoBIOMICS™ Microbial Community DNA Standard (D6305) and an internal 16S mock bacterial community control (DNA QC 16S) from 10 genomic DNAs obtained from DSMZ (Lot No: 2019-1) were also run in quadruplicate. Libraries were prepared using Quick-16S™ NGS Library Prep Kit (Zymo Research, Irvine, CA), according to the manufacturer protocol. The final pooled library was quantified with Qubit^®^ and the amplicons were sequenced on an Illumina MiSeq with MiSeq^®^Reagent Kit v2 (500-cycle) (Illumina, USA). The raw sequencing data have been deposited in the European Nucleotide Archive (ENA) at EMBL-EBI under the study accession number PRJEB45381. Sequences were processed with the program mothur (v1.44.3) [41] according to the MiSeq SOP, which can be found on the mothur website (https://mothur.org/wiki/miseq_sop/, accessed on 23 June 2021) [40,42].

### 4.6. Intestinal Fatty Acid Analysis

Thirty to one hundred mg of flash-frozen cecal content was homogenized using 1.4 mm ceramic beads (5 beads per tube). Per 50 mg of cecal content, 500 µL of stock solution (2-Ethylbutyric acid, 20 mmol/L) was used (VK05 Tough Micro-Organism Lysing Kit). Cecal content was homogenized for 30 sec at 4500× *g* at 10 °C (Precellys24 Homogenizer) and centrifuged at 21,000× *g* for 5 min and 4 °C. Sample homogenate was further processed and measurements of SCFAs was performed as previously described using high-performance liquid chromatography (HPLC) [43].

### 4.7. Detection of Glycan-Degrading Enzyme Activities in Fecal Samples

Enzymatic activities of β-glucosidase, α-galactosidase, α-fucosidase, β-*N*-acetylglucosaminidase and sulfatase were detected from fecal samples stored at −20 °C as described previously [44]. In brief, bacterial glycan-degrading enzymes were solubilized from the fecal samples by incubation in a lysozyme-, DNase I- and Triton X-100-containing lysis buffer on ice followed by sonification and removal of unsolubilized material by centrifugation. Supernatants were collected and protein concentrations in these supernatants was measured. For detection of enzymatic activities, equal amounts of protein were incubated with enzyme-specific *p*-nitrophenol-coupled substrates, and the substrate turnover (*p*-nitrophenol release) was monitored by kinetic measurements of optical density at 405 nm. For details on buffers, substrates and final computation of enzymatic activities from optical density data, refer to [44].

### 4.8. Data Analysis

Data transformation and analysis was performed in *R Studio*, version 4.0.2 (22 June 2020) using the following packages: readxl (version 1.3.1) [45], ggplot2 (version 3.3.3) [46], ggpubr (version 0.4.0) [47], ggfortify (version 0.4.11) [48], Hmisc (version 4.5.0) [49], xfun (version 0.23) [50], corrplot (version 0.84) [51], corrr (version 0.4.3) [52] and stats (version 4.0.2). For comparisons of non-normally distributed data sets, a non-parametric Wilcoxon rank sum test was used with a significance level of 0.05. For pairwise comparison of two groups with normally distributed values, a parametric *t*-test was used with a significance level of 0.05.

## Figures and Tables

**Table 1:** Composition of the used concentrated fiber preparations in the FS diet.

Origin of Fiber Preparation	Total Dietary Fiber Content	I/S Ratio	pH Value (10% Solution)	Pectin Content	Oxide Ash	Bulk Density	Average Fiber Length
Pea	~70%	~34	4.0–7.0	N/A	max. 5%	300–620 g/L	N/A
Oat	~90%	N/A	5.5–7.5	N/A	max. 5%	260–385 g/L	75 µm
Psyllium	~80%	~0.2	5.0–7.0	N/A	max. 3%	350–700 g/L	N/A
Wheat	~97%	N/A	5.0–8.0	N/A	max. 3%	260–355 g/L	50 µm
Apple	~55%	~4.5	3.0–5.0	~9%	max. 3%	255–345 g/L	N/A

N/A = information not available; I/S Ratio = Proportion of insoluble fiber compared to proportion of soluble fiber within the total dietary fiber content of the preparations.

## Data Availability

The raw sequencing data have been deposited in the European Nucleotide Archive (ENA) at EMBL-EBI under the study accession number PRJEB45381.

## Abbreviations

AA	Amino acids
AM	*Akkermansia muciniphila*
BC	*Bacteroides caccae*
BI	*Barnesiella intestinihominis*
BO	*Bacteroides ovatus*
BT	*Bacteroides thetaiotaomicron*
BU	*Bacteroides uniformis*
CA	*Collinsella aerofaciens*
CRF	Concentrated raw fiber
CS	*Clostridium symbiosum*
DP	*Desulfovibrio piger*
EC	*Escherichia coli*
ER	*Eubacterium rectale*
FF	Fiber-free
FP	*Faecalibacterium prausnitzii*
FS	Fiber-supplemented
FUC	α-Fucosidase
GAG	Glucosaminoglycans
GAL	α-Galactosidase
GF	Germ-free
GLUC	β-Glucosidase
HPLC	High-performance liquid chromatography
I/S-ratio	Insoluble-to-soluble fibers
MF	*Marvinbryantia formatexigens*
MS	Mono-saccharides
MVA	Minerals, vitamins, and ash
NAG	β-*N*-Acetylglucosaminidase
NFE	Nitrogen-free extracts
PCA	Principal components cnalysis
RI	*Roseburia intestinalis*
SC	Standard mouse chow
SCFA	Short-chain fatty acid
SM	Synthetic microbiome
SULF	Sulfatase
